# An Integrated Approach to Improve Maternal Mental Health and Well-Being During the COVID-19 Crisis

**DOI:** 10.3389/fpsyt.2020.598746

**Published:** 2020-11-24

**Authors:** Rahul Shidhaye, Purnima Madhivanan, Pallavi Shidhaye, Karl Krupp

**Affiliations:** ^1^Directorate of Research, Pravara Institute of Medical Sciences, Loni, India; ^2^Department of Psychiatry, Pravara Institute of Medical Sciences, Loni, India; ^3^Department of Health Promotion Sciences, Mel and Enid Zuckerman College of Public Health, University of Arizona, Tucson, AZ, United States; ^4^Division of Infectious Diseases and Department of Family and Community Medicine, College of Medicine, University of Arizona, Tucson, AZ, United States; ^5^Public Health Research Institute of India, Mysore, India; ^6^Indian Council of Medical Research-National AIDS Research Institute, Pune, India

**Keywords:** pregnancy, maternal mental health, self-care (MeSH), social support (MeSH term), health system, COVID-19 pandemic

## Abstract

The ongoing COVID-19 pandemic has led to disruption of normal life across the globe, severely affecting the already vulnerable populations such as the pregnant women. Maternal mental health and well-being is a public health priority and the evidence about the impact of COVID-19 on mental health status of pregnant women is gradually emerging. The findings of the recently published studies suggest that increased risk perception about contracting COVID-19, reduced social support, increase in domestic violence, disruption of antenatal care, and economic consequences of COVID-19 mitigation strategies can lead to adverse mental health outcomes in antenatal period. There is a significant increase in antenatal depression and anxiety since the onset of COVID-19 and social determinants of health (e.g., younger age, lower education, lower income) are associated with these poor outcomes. In this paper, we propose an integrated approach to improve the mental health and well-being of pregnant women. Physical activity and/or mind-body interventions like yoga can be practiced as self-care interventions by pregnant women. Despite social distancing being the current norm, efforts should be made to strengthen social support. Evidence-based interventions for perinatal depression should be integrated within the health system and stepped, collaborative care using non-specialist health workers as key human resource be utilized to improve access to mental health services. Use of digital platforms and smartphone enabled delivery of services has huge potential to further improve the access to care. Most importantly, the COVID-19 related policy guidelines should categorically include maternal mental health and well-being as a priority area.

## Introduction

In just over 6 months, the COVID-19 pandemic has upended lives across the world. Governments in most of the countries have imposed varying levels of “lockdowns” and requested citizens to “stay at home” to contain the spread of the virus and prevent health systems from being overrun. The resultant closure of workplaces, schools, day-care centers, and healthcare services has disrupted almost every aspect of life-with disastrous consequences especially for the vulnerable sections of the population such as adolescents, elderly, women, people living in poverty, and those with severe mental health conditions (1). Pregnancy also increases vulnerability as women undergo changes in physical and psychological functioning throughout gestation, labor, and delivery. Recent data suggests that pregnant women may be at elevated risk for COVID-19 infection and hypoxic compromise due to changes in the cardiorespiratory and immune function (1), and a previous systematic review found that the pregnant women had significantly higher rates of mood disorders during disasters compared to the general population (2). In low-and-middle income countries (LMIC), antenatal depression is already a major public health problem with a prevalence of 25.3% (95 confidence interval [CI]: 21.4–29.6%) (3). Severe disruption of life due to COVID-19, fear of contracting the disease, and anticipation of negative economic consequences have led to increased symptoms of depression, anxiety, and stress in the general population as well as in the health care providers (4). However, a rapid evidence review and two other editorials did not find any studies on the impact of COVID-19 on maternal mental health and well-being (5–7). Evidence in this domain is gradually emerging and in this perspective paper, we provide a high-level overview of the recently published epidemiological studies assessing the impact of COVID-19 on maternal mental health, and propose an integrated approach to improve mental health and well-being of pregnant women during the current crisis. A comprehensive search strategy was designed to identify the evidence; however, the search was limited only to the Pubmed. The details about the search strategy, screening, and selection of published articles are provided in Box 1.

Box 1 **Search strategy and study selection.**

In order to understand the impact of COVID-19 on maternal mental health, we searched PUBMED using the terms, (((pregnancy OR pregnant OR pre-nat^*^ OR prenat^*^ OR prepart^*^ OR prepart^*^ OR ante-nat^*^ OR antenat^*^ OR ante-part^*^ OR antepart^*^ OR peri-nat^*^ OR perinat^*^ OR peri-part^*^ OR peripart^*^ OR puerper^*^ OR post-nat^*^ OR postnat^*^ OR post-part^*^ OR postpart^*^)) AND (mental^*^ OR psych^*^ OR anxiety OR anxious OR depress^*^ OR mood? OR affect^*^ OR distress^*^ OR stress or trauma^*^ OR posttrauma^*^ OR post-trauma^*^ OR adjustment disorder^*^ OR phobia^*^ OR phobic OR obsessive compulsive OR wellbeing OR well-being)) AND (coronavir^*^ OR coronovirus^*^ OR “corona virus” OR “virus corona” OR “corono virus” OR “virus corono” OR hcov^*^ OR “covid-19” OR covid19^*^ OR “covid 19” OR “2019-nCoV” OR cv19^*^ OR “cv-19” OR “cv 19” OR “n-cov” OR ncov^*^ OR “sars-cov-2” OR (wuhan AND (virus OR viruses OR viral OR coronav^*^)) OR (covid^*^ AND (virus OR viruses OR viral)) OR “sars-cov” OR “sars cov” OR “sars-coronavirus” OR “severe acute respiratory syndrome)

The search was carried out from 15 July to 23 July (the last date of our search) and all the articles in English language were included. Our search identified a total of 1010 citations which were imported in Mendeley. One author (PS) screened the titles and abstracts using Rayyan software. Full text of 26 articles was screened and 11 articles were found to be relevant. Forward and backward searches of included studies were carried out and two articles were additionally included.

## The Intersection of COVID-19 and Maternal Mental Health

COVID-19 can impact maternal mental health in following ways:

First, there is increased **risk perception** about contracting COVID-19, which can directly increase maternal anxiety. In China, pregnant women living in the epidemic center (Wuhan city) and those experiencing subjective symptoms were far more likely to have anxiety than otherwise healthy pregnant women (8). A study of pregnant women attending tertiary care center in Turkey reports that 80% of the respondents were overwhelmingly distressed about the pandemic and half of them (52%) felt vulnerable due to their pregnancy. The respondents were also preoccupied about getting infected during/following the delivery (35.5%) or that their baby may get the infection after being born (42%) (9). Infection of other family members was perceived as a bigger concern in pregnant women in Israel than being infected themselves (71.7 vs. 59.2%) (10). Half of the pregnant women interviewed during ante-natal care visit in Ireland, expressed worry about their health often or all the time. They had heightened anxiety about the health of their older relatives (83.3%), concerns about their children (66.7%), and their unborn baby (63.4%) (11).

Second, COVID-19 mitigation strategies have restricted movement and transportation, socialization and engagement in normal routines leading to isolation and **reduced social support**. There are a range of traditional practices associated with pregnancy and birth in various cultures across the world such as *Seemantham* and *Garbhasanskaras* in India (12), *Tsao-Yueh-Tzu (*“*doing the month*”*)* in China (13), *Sam chil il* in Korea, *la cuarenta* in Mexico, and so on (14). Pandemic mitigation strategies have led to disruption of several of these activities which can potentially impact the mental health of pregnant women. In Asian countries, women often return to their parent's home for the delivery and remain there for several months after the delivery for nurturing care of the baby. In Japan this practice is called *Satogaeri* and the restrictions by the Japanese government on the same may lead to adverse maternal mental health outcomes (15). Another Japanese study also documents that concerns about social support were significantly associated with perceived stress in pregnant women (16). Lack of support in child-care was expressed as a major concern by pregnant women from other parts of the world as well (11, 17).

Third, staying at home increases the risk of **intimate partner violence** (IPV) for women who are in abusive relationships (18). Globally, 30% of women experience physical or sexual violence by an intimate partner in their lifetime (19). Such violence typically increases in frequency and severity during pregnancy (20) and humanitarian crises and natural disasters (18). Recent reports indicate that physical distancing measures may limit the ability of victims of violence to distance themselves from abusers or access external support (21). The evidence suggests that ongoing IPV has a substantial effect on a woman's physical and mental health, especially during the antenatal period (22).

Fourth, serious disruption in the **provision of antenatal care** can further increase the treatment gap for maternal mental disorders. The health systems, especially in the LMICs are extremely stretched due to the pandemic. The availability of sexual and reproductive health services in the Asia Pacific region will decrease by about one fifth in the best scenario, and by half in the worst scenario according to the World Economic Forum (23). The reasons are several. Pregnant women have been reluctant to visit the health facilities during the pandemic (8) due to concerns related to use of public transportation and exposure to COVID-19 during the check-up visit (10). On the other hand, health care providers have been minimizing non-essential obstetric visits, excluding birth partners during labor and birth, separating mother and baby in the immediate postnatal period and restricting breastfeeding ultimately affecting the overall quality of care (21, 24). Mental health is generally accorded low priority in LMIC health systems and with the current crisis the situation will worsen further.

Fifth, the medium- and long-term **economic consequences** of COVID-19 and the financial uncertainties will escalate the psychological burden and worsen the mental well-being of pregnant women and new mothers. This may be particularly harmful for women in low socio-economic class as they constitute high risk and vulnerable group for antenatal depression. In a cross-sectional study from Japan, pregnant women expressed concerns about future expenditure related to delivering the baby and the nursing care. These concerns about future household finances were associated with increased maternal stress (16). Approximately two-thirds of the pregnant women respondents in the US survey reported stress about losing a job or loss of household income (63.7%) (17), while in Israel the proportion of pregnant women feeling anxious about the possible economic damage of the pandemic was much less (38.1%) (10). Global recession because of the pandemic will likely lead to job loss, reduced income, and most importantly disruption of food supply chains leading to Household Food Insecurity (HFI) (25). Research evidence suggests that food insecurity is negatively associated with the mental health of mothers and has negative impact on early child development outcomes (26).

## Effect of COVID-19 on Maternal Mental Health

Several studies from different parts of the world published in last 3 months have now assessed the impact of COVID-19 situation on mental health outcomes in pregnant women.

### Mental Health Status Pre-COVID-19 and During COVID-19

Two studies, one from China (27) and other from Canada (28) compared mental health outcomes in pregnant women recruited before and after the onset of COVID-19. The Chinese study recruited a total of 4,124 pregnant women from 10 different provinces to assess the impact of COVID-19 on the prevalence of depression and anxiety and the risk factors associated with these outcomes (27). Pregnant women assessed after the declaration had higher prevalence of depressive symptoms (29.6 vs. 26.0%), and this increase of 3.4% was statistically significant (*p* = 0.2). They were more likely to have thoughts of self-harm. A linear positive correlation was found between the prevalence of depression and the number of newly confirmed cases of COVID-19, suspected infections, and deaths per day (27). Similar findings were reported from Canada. Cohort of pregnant women recruited after the onset of COVID-19 were twice likely to have clinically significant symptoms of depression and anxiety than those recruited prior to COVID-19 (10.9 vs. 6.0%) (28). They also had higher levels of symptoms of post-traumatic disorder, dissociative symptoms, negative affectivity, and less positive affectivity (28).

Increase in psychological distress during COVID-19 is also supported by other cross-sectional studies. The prevalence of self-identified depression and anxiety more than doubled in a large sample of Canadian pregnant women who completed an online survey. Moderate to high anxiety was identified in 29% of women before the pandemic compared to 72% of women after the pandemic started while 15% of the respondents had depression pre-pandemic compared to 40.7% during the pandemic (29). An assessment of Italian pregnant women using State-Trait Anxiety Inventory (STAI) found that there was doubling of state anxiety scores compared to trait anxiety scores (30). An online survey of 5,866 women in Belgium (2,421 pregnant women) found that almost half of the women experienced depressive or anxious symptoms during the lockdown period, and the prevalence of self-reported major depressive symptoms was 25.3% and 14% met the criteria for high anxiety. This was higher compared to estimates obtained in Belgium prior to the pandemic (31). Psychological impact of COVID-19 was severe in more than half of the pregnant women in Italy while a third of the pregnant women (35.4%) were screened positive for depression in Turkey (32, 33).

### Risk Factors Associated With Mental Health Outcomes

Cross-sectional survey of 2,740 pregnant women from 47 states in the United States found that younger age, advanced pregnancy (third trimester), previous history or recent diagnosis of depression/anxiety, and being an essential worker (or family member an essential worker) were associated with increase in scores on Pregnancy Related Anxiety Scale (PRAS). Pregnant women residing in areas with high number of COVID-19 infections, those experiencing stress about availability of food and conflict at home also reported higher anxiety scores (17). Similar factors such as younger age, lower education, lower household income, and history of a psychiatric disorder were associated with psychological distress in the Canadian survey of pregnant women (28). The evidence from China about the association of income and mental health outcomes is equivocal. Liu et al., found that pregnant women from middle-level income families were less likely to report anxiety than those from low or high-income families (8). On the other hand, according to Wu et al., pregnant women from the middle-income category, and working full time were more likely to experience depressive symptoms (27). In the US, women in the third trimester had higher anxiety scores (17) while in Italy, one study found that women in the first trimester of pregnancy were more affected (32), but another study from Italy found no association with gestational age (30). Residence was another important factor associated with mental health outcomes. Pregnant women in Wuhan, the epicenter of this global pandemic, had twice the odds of developing anxiety (OR: 1.83, 95% CI 1.38–2.41) compared to the pregnant women in Chongqing (less affected city in China) (8).

## 5S Approach to Improve Maternal Mental Health and Well-Being

Till now, the papers published on the topic of maternal mental health and COVID-19 are either cross-sectional epidemiological studies or editorials/commentaries. We did not find any publication which provides an approach to improve maternal mental health and well-being. We try to address this gap through the “***5S***” approach described below. This approach is based on our previous work related to improving mental health services, included in the mental health volume of Disease Control Priorities-3 (34). We now modify the health care platforms-based approach described earlier to address the issue of maternal mental health in the current pandemic. The approach described below is also supported by the findings in the recently published papers including a qualitative study which assessed the factors related to resilience in pregnant women (35).

**Figure 1:**
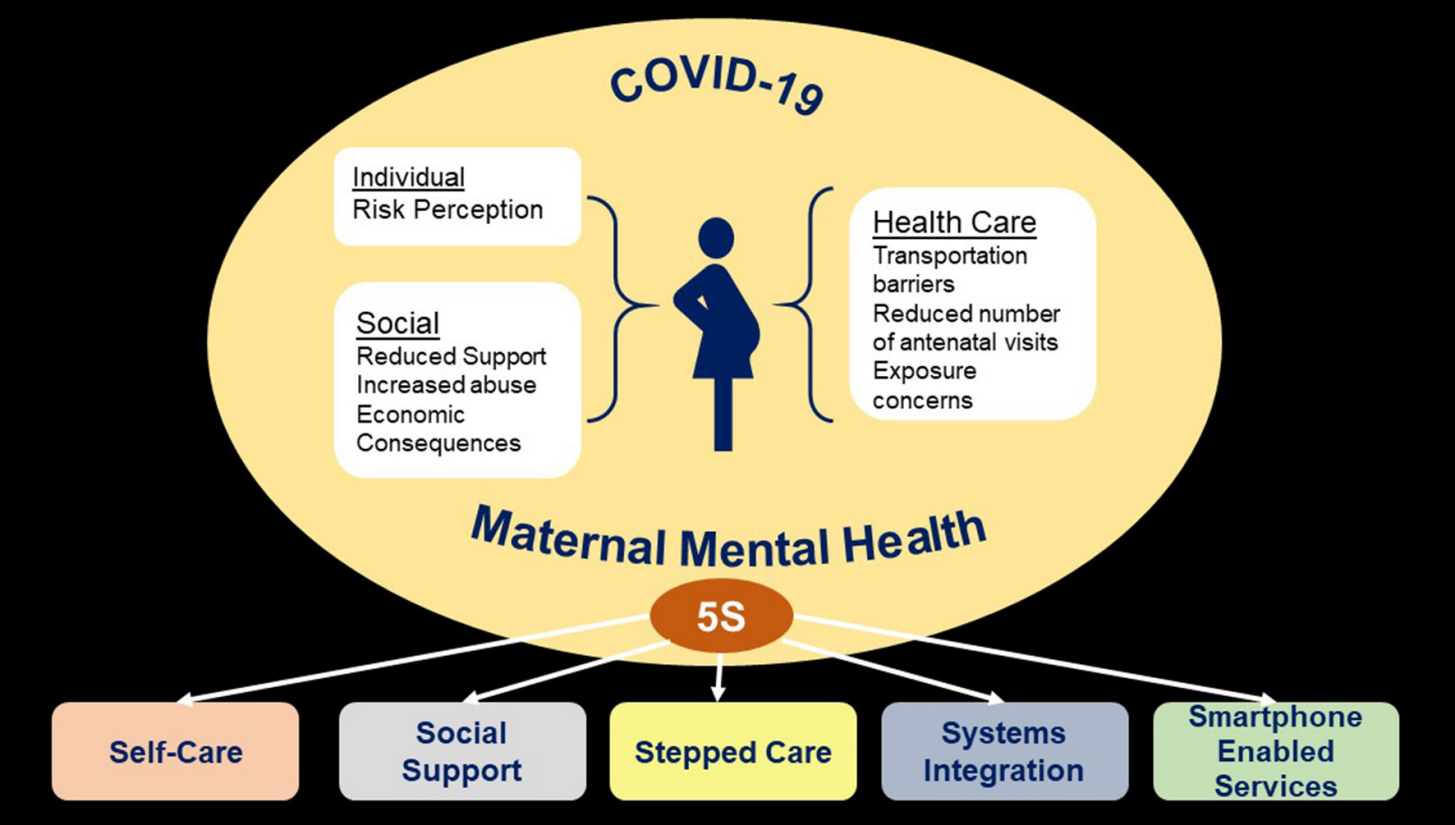
5S approach for maternal mental health in COVID-19 crisis.

The 5S approach comprises of Self-Care, Social Support, Stepped Care, Systems Integration, and Smartphone enabled services (Figure 1).

### Self-Care and Mind-Body Interventions

In order to achieve the goal of Universal Health Coverage, Self-Care is becoming increasingly important with half of the world's population (3.6 billion) having little or no access to needed health services and the World Health Organization (WHO) estimating a shortage of nearly 13 million mental healthcare workers by 2035 (36). There is growing support for self-care management and informal community mental health services as “base-of-the-pyramid” interventions' (37, 38).

During the current COVID-19 crisis, home-based physical activity, mind-body interventions such as yoga, mindfulness, and relaxation exercises can be practiced by pregnant women as Self-Care to improve their mental health and well-being. They are easy to learn and use in self-isolation and lock-down contexts (6). Davenport et al., found that pregnant (and post-partum) women engaging in at least 150 min of moderate intensity physical activity per week during the pandemic had significantly lower scores for both anxiety (large effect) and depression (small effect) than those who did not (29). Physical activity was also independently associated with depression in a study by Wu et al. (27), as a result they recommend programs to support exercise/physical activity across the perinatal period.

Recently published meta-analysis found that yoga compared with treatment as usual/waitlist control had positive impact on improving depression outcomes with a standardized mean difference of −0.41 (95% CI −0.65 to −0.17) (39). Thus, physical activity and yoga can be potentially used as Self-Care interventions by pregnant women. In the current situation, closure of indoor recreation centers, yoga studios, gymnasiums, and outdoor parks/greenspace are critical barriers to practice. Activities such as gardening, going for walks while maintaining physical distance, household chores, home-based practice of yoga, and online exercise classes are some feasible alternatives. They can be promoted to increase physical activity in pregnant women (29).

### Social Support

During the current times when “social distancing” has become the new normal, the importance of social support has increased. Social support was found to play an important role in reducing antenatal anxiety in a cross-sectional study of 308 Chinese pregnant women. Social support reduced antenatal anxiety directly as well as indirectly by reducing the risk perception (40). Supportive networks are of central importance to maternal mental health and can still be engaged with virtually during the COVID-19 pandemic. Health care providers can actively mobilize the social support system for pregnant women by encouraging them to maintain contact with loved ones and referring them to support groups and other social services coordinated by the Non-Governmental Organizations. Through health education programs they can reduce the risk perception level of pregnant women in relation COVID-19. In a study from a rural low-resource setting in India, we found that pregnant women more frequently visited by community health workers at home and more frequently accompanied by them to antenatal care visits were less likely to report anxiety during pregnancy (41). In low resource settings with overburdened public health system, Non-Governmental Organizations can play an important role in providing support to pregnant women through integrated community-based activities focusing on reducing household food insecurity and improving social support to improve maternal mental health.

### Stepped Collaborative Care

Stepped care approach involves delivery of low-intensity, low-cost interventions to patients as a first step and only if necessary, they move to higher-intensity treatment. Stepped care is usually combined with collaborative care which brings together different care providers to improve quality of care, satisfaction of patients and the system efficiency (42). Collaborative care has been used successfully for the management of common mental disorders such as depression and various other co-morbid conditions across a range of settings have been successfully managed by the collaborative care approach (43).

The bedrock of stepped collaborative care is delivery of low intensity psychosocial interventions by the non-specialist health workers. A number of such interventions have been developed and evaluated for treatment of perinatal depression in LMICs (44). Although none of these interventions have been developed for mental health crisis during pandemic, they can be adapted for a particular context and setting. The Thinking Health Program (45) is one such intervention and has been successfully evaluated in Pakistan (46) and India (47). The intervention is delivered by the community health workers or peers in the community and pregnant women not showing improvement are referred to primary care physicians and/or specialists for further management. The challenges in delivering interventions by the non-specialist health workers include lack of financial resources, infrastructure for training, providing continuing clinical supervision to non-specialist health workers, difficulty in retaining these workers in the absence of compensation/incentives, high workloads, logistic barriers related to scheduling follow-up appointments and transportation costs (48). These challenges can be addressed by health systems strengthening and using an implementation research approach as described below.

### Systems Integration

Interventions proven to be effective in a controlled setting of a randomized trial, often fail to deliver outcomes in a routine practice (49). This may not be due to any problem in the intervention itself, but rather the unpredictability of the system around it (50). In an unmapped and misunderstood health system, even a simple intervention fails. The evidence-based interventions described above should be adapted for the specific culture and the health system context before they are implemented (51). In addition to the training and clinical supervision of the health care providers, facilitation by the support team (52) using a range of implementation strategies (53) can be helpful in mitigating the challenges associated with the delivery of mental health services.

One potential way to integrate maternal mental health services in the existing health systems is by providing psychological support to midwives and other health care providers working on the frontline who are likely to suffer from moral injury (54), burnout, and other mental health problems (55) due to major changes in patient management. Measures to improve mental health of frontline workers and health care providers may lead to positive attitudes toward mental disorders among the staff members and establish an enabling environment to integrate mental health services in the maternal and child health care system (24).

### Smartphone Enabled Services

The current pandemic provides us an opportunity to revolutionize the use of smartphone to deliver digital mental health. It has the potential to scale up the delivery of mental health services to patients across a wide range of platforms, from tele-mental health to smartphone-based interventions such as apps and text messaging (56). Tele-health through video-conferencing is as effective as in-person service provision and researchers in China successfully provided online psychological counseling and self-help in medical institutions and universities (56). However, telehealth and digital services should not replace face-to-face treatment for patients in need, particularly those requiring intensive mental health treatment and support when “in-person” contact is once again safe. All pregnant women in low resource settings may not have access to digital platforms, especially those in the lower quintiles of socioeconomic strata. The inequitable access to care will be further widened if the face-to-face services are extensively shifted to digital platforms and due to this “*digital divide*,” women with social vulnerabilities may suffer the most. Another critical aspect is to ensure the safety of women who are already experiencing domestic violence. Use of mobile phones may endanger their health and safety, if the abuser finds out that they are looking for help or reporting abuse.

## Implications for Research, Practice, and Policy

In the general population, social determinants of health such as female gender, low education, lower household income, and low level of social capital are associated with the depression and anxiety due to COVID-19 pandemic (4). The current evidence however, is unable to inform us of the risk factors in pregnant women for adverse mental health outcomes. As a result, it is critical to collect data on social determinants of health to effectively address COVID-19 related health inequities. The other important research gap is related to lack of data from the LMICs. Except for a few, most of the studies are from high income countries. We also need to understand the impact of poor maternal mental health due to COVID-19 on the birth outcomes, neonatal care including exclusive breastfeeding and duration of breastfeeding, and immunization rates. If the pandemic continues for long it can adversely affect early childhood development. Interventions which have proven to be effective in improving maternal mental health need to adapted for the current situation and their effectiveness in the “new normal” needs to be assessed. Efforts should also be made to integrate evidence-based mental health interventions in routine maternal health care. Finally, the policy documents and guidelines issued by the health ministries and other agencies should pay adequate attention to improve physical as well as mental health and well-being of pregnant and postnatal women.

## Data Availability Statement

The original contributions presented in the study are included in the article/supplementary material, further inquiries can be directed to the corresponding author.

## Author Contributions

RS: conceptualization, methodology, formal analysis, data curation, and writing-original draft. PM: conceptualization, writing-review, and editing. PS: methodology, formal analysis, data curation, writing-review, and editing. KK: writing-review and editing. All authors read and approved the final draft of the paper. All authors contributed to the article and approved the submitted version.

## Conflict of Interest

The authors declare that the research was conducted in the absence of any commercial or financial relationships that could be construed as a potential conflict of interest.
